# Evaluating the Acceptability and Pilot Diagnostic Accuracy of a Visually Independent Test Battery of Neurocognition (VISION-Cog)

**DOI:** 10.3390/medsci14030344

**Published:** 2026-06-24

**Authors:** Hiromi Yee, Aricia Xin Yi Ho, Chiew Meng Johnny Wong, Wei Lin Tan, Eva K. Fenwick, Preeti Gupta, Adeline S. L. Ng, Tai Anh Vu, Kinjal Doshi, Ecosse L. Lamoureux, Ryan E. K. Man

**Affiliations:** 1 Singapore Eye Research Institute (SERI), Singapore National Eye Centre, The Academia, Singapore 169856, Singapore; hiromi.yee1@alumni.lshtm.ac.uk (H.Y.); aricia.ho.x.y@seri.com.sg (A.X.Y.H.); johnny.wong.c.m@seri.com.sg (C.M.J.W.); tan.wei.lin@seri.com.sg (W.L.T.); efenwick@duke-nus.edu.sg (E.K.F.); preeti.gupta@duke-nus.edu.sg (P.G.); ryan.man@duke-nus.edu.sg (R.E.K.M.); 2 Duke–NUS Medical School, National University of Singapore, Singapore 169857, Singapore; adeline.ng.s.l@singhealth.com.sg (A.S.L.N.); vu.taianh@u.duke.nus.edu (T.A.V.); 3 National Neuroscience Institute, Tan Tock Seng Hospital, Singapore 308433, Singapore; 4 Department of Psychology, National University of Singapore, Singapore 117572, Singapore; kinjal@insightconsult.com.sg; 5 Department of Ophthalmology, University of Melbourne, Parkville Victoria 3010, Australia

**Keywords:** cognitive impairment, visual impairment, cognitive assessment, neuropsychology

## Abstract

Background: Cognitive impairment (CI) may be overdiagnosed in individuals with vision impairment (VI) due to the vision-dependent design of current cognitive assessment tools. This cross-sectional study evaluated the acceptability and diagnostic accuracy (pilot) of the Visually Independent Test Battery of Neurocognition (VISION-Cog) protocol, against gold-standard neurologist diagnosis. Methods: Community-dwelling older adults with near binocular presenting VI (near visual acuity [NVA] ≥ 0.2 logarithm of the minimum angle of resolution [LogMAR] units) were recruited from the Population Health and Eye Disease Profile in Elderly Singaporeans (PIONEER) study. Participants underwent VISION-Cog and the Singapore-validated Montreal Cognitive Assessment (MoCA-SG) testing and were referred for neurologist evaluation based on standardized referral protocols. The acceptability of the VISION-Cog was assessed through study completion rates, test duration, and the qualitative feedback. Vision-Cog’s diagnostic accuracy (pilot) against neurologist evaluation was analyzed using binary logistic regression and C-statistics to estimate area under the receiver operating curve (AUC) with corresponding sensitivity and specificity. Results: Out of forty-five participants (mean age [SD]: 73.8 [6.1 years]; mean NVA [SD]: 0.47 [0.14] LogMAR; and 54.1% female), 37 (82.2%) completed the protocol. The mean VISION-Cog completion time [SD] was 59 m 57 s (7 m 18 s). Qualitatively, participants found the testing time acceptable. The VISION-Cog achieved an AUC of 0.930 against neurologist diagnosis, with 100.0% sensitivity and 78.0% specificity. Conclusions:The VISION-Cog demonstrated satisfactory preliminary diagnostic accuracy and good acceptability indices in older Asian adults, supporting the need of larger studies to confirm its diagnostic accuracy of CI and clinical utility in those with VI.

## 1. Introduction

The concomitant occurrence of visual and cognitive impairment (VI and CI, respectively) is expected to rise exponentially as the population ages [1]. Currently, one in every three adults with CI are estimated to be visually impaired [2]. In Asia specifically, where the population of individuals aged 65 years and older is projected to exceed 1.3 billion by 2050 [3], the compounding effect of VI and CI is expected to place significant strain on neurological and ophthalmic care systems alike.

Cognitive screening instruments (e.g., the Montreal Cognitive Assessment [MoCA]) and neuropsychological test batteries (e.g., the Repeated Battery for the Assessment of Neuropsychological Status [RBANS]) are used to facilitate the diagnosis of CI [4]. However, these tests rely heavily on vision [5], which may compromise cognitive assessment in those with VI, leading to potential overestimation of CI, particularly mild CI (MCI), in these individuals [6]. Indeed, previous studies have shown that patients with VI due to cataracts or age-related macular degeneration achieved poorer scores on vision-dependent cognitive tasks compared to those without VI [7,8]. Importantly, it has been established that patients with VI perform better on visually independent cognitive tests as opposed to equivalent vision-dependent cognitive assessments [9]. Given the prodigious consequences of CI on patients, caregivers, and society, valid and reliable visually independent assessment tools that can accurately diagnose CI are essential so that appropriate healthcare resources may be designated for the clinical management of older individuals with CI.

Several CI assessment tools have been adapted for visually impaired individuals by removing vision-related questions. For example, the MMSE-blind was designed by removing eight vision-dependent tasks in the language and visuospatial domains [10] while the MoCA-blind was developed by omitting four vision-dependent items in the visuospatial, executive, and naming domains, including the trail-making, copy-cube, clock-drawing, and picture-naming tasks [11]. However, the internal validity of these amended tests compared to their original versions is questionable due to inferior diagnostic indices consequent to item deletion [5]. Indeed, the sensitivity in detecting MCI of the MoCA-blind is only 44%, much lower than that of the original MoCA version [11]. In addition, to the best of our knowledge, neuropsychological batteries created for visually impaired patients such as the Haptic Intelligence Scale, the Cognitive Test for the Blind, and the Vision Independent Cognitive Screen are either no longer commercially available or still under development [12]. The lack of a standardized cognitive assessment protocol for individuals with VI often results in the overdiagnosis of CI among visually impaired older adults. This issue is particularly prevalent in settings where expertise in neuropsychological assessments is limited, such as specialized memory clinics (MCs) within primary care, where patients with suspected CI are referred by primary care physicians for further evaluation and monitoring. Moreover, even in tertiary neurological care with experienced clinical psychologists, current practice relies on ad hoc modifications by these psychologists, creating a lack of standardization across assessments conducted by these practitioners.

To address these limitations, we developed and validated the Visually Independent Test Battery of Neurocognition (VISION-Cog) in collaboration with neurologists, neuro-ophthalmologists and neuropsychiatrists, via a series of iterative pilot studies and expert panel discussions [13]. This five-domain neuropsychological test battery incorporates tactile- and auditory-based assessments to evaluate the presence of CI in older adults with VI [13]. However, there is a lack of data concerning the acceptability of administering the VISION-Cog to its intended population, which comprises individuals with VI with and without CI, nor is there any information on its diagnostic performance relative to evaluation by a clinical neurologist. In this pilot study, we aimed to evaluate the acceptability of the VISION-Cog protocol in terms of study completion rates and assessment length, supplemented by qualitative feedback from participants, caregivers and the VISION-Cog administrator. Secondarily, the study aimed to determine the diagnostic accuracy (pilot) of VISION-Cog in detecting CI in this population, compared to gold-standard neurologist diagnosis. We hypothesize that VISION-Cog will have good study completion rates and comparable completion time to existing neuropsychological batteries and demonstrate high discriminative accuracy (area under the receiver operating characteristic curve [AUC] ≥ 0.8) in detecting those with and without CI compared to clinical neurological evaluation. These data will inform the design of a larger prospective study to validate the clinical effectiveness of the VISION-Cog in detecting CI in visually impaired patients relative to both existing vision-dependent neuropsychological test batteries and clinical neurologist diagnosis.

## 2. Methods

### 2.1. Study Population

This cross-sectional clinical study was conducted at the Singapore National Eye Centre with participant recruitment taking place between Jan 2024 and March 2025. Participants with near VI, previously enrolled in the baseline phase of the community-based cohort study Population Health and Eye Disease Profile in Elderly Singaporeans (PIONEER), were recruited [14]. English- and/or Mandarin-speaking participants were purposively recruited across a range of ages, genders, education, and VI levels, based on sociodemographic and clinical data available within PIONEER. All participants provided written informed consent before participation and the study adhered to the Declaration of Helsinki, with ethics approval from Singapore’s Centralized Institutional Review Board (CIRB# R1968/11/2023). Individuals with clinically diagnosed dementia, insufficient cognitive capacity to provide consent, or severe hearing or tactile impairments were excluded. The STARD reporting guidelines and checklist were used to draft this manuscript [15].

### 2.2. The VISION-Cog Assessment Protocol

The VISION-Cog is a novel neuropsychological instrument designed to evaluate the presence and severity of CI in visually impaired older adults using 9 vision-independent tests across 5 distinct neurocognitive domains critical to CI classification within the Diagnostic and Statistical Manual of Mental Disorders, 5th Edition (DSM-5) [16], including Memory and Learning, Language, Executive Function, Complex Attention, and Perceptual-Motor. The development of the VISION-Cog comprised two key phases. In the content development phase, the content of a pilot VISION-Cog was generated from literature reviews and consultation with field experts including psychologists, geriatricians, neurologists, neuro-ophthalmologists, and psychiatrists. Thereafter, pilot testing of the VISION-Cog was undertaken to fine-tune its content via iterative pilot studies and expert panel discussions to form a final revised version. In this study, the VISION-Cog was administered in a quiet room in English or Mandarin depending on participants’ preference by a single trained research associate. To ensure clear communication and facilitate participant responses, a microphone was employed to amplify test instructions, especially for individuals with hearing impairment. Testing was done with participants’ habitual correction, and the administration time was recorded using the stopwatch function of a Samsung Galaxy A10s smartphone (Samsung Electronics Co., Ltd., Suwon-si, Republic of Korea).

### 2.3. Visual and Other Cognitive Assessments

**Figure 1:**
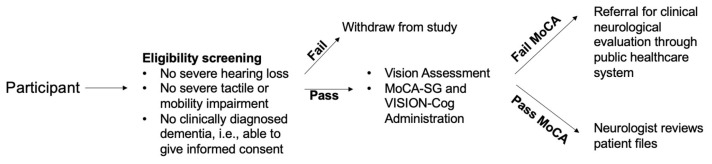
Flow diagram of clinical protocol.

The habitual binocular presenting near visual acuity (VA) of participants was assessed using a logarithm of the minimum angle of resolution (LogMAR) reading chart at 40 cm under standard lighting conditions (85 cd/m^2^), with near VI defined as VA > 0.2 LogMAR [17]. All participants were administered the Singapore-validated MoCA (MoCA-SG) [18]. Participants who failed the MoCA-SG (score <26 and <27 for individuals with ≤10 years and >10 years of education, respectively) [18] were referred for further clinical neurologist evaluation through the publicly funded healthcare system. The neurological evaluation followed standardized protocol: a clinical neurologist would review the individual’s MoCA-SG score and case history, supplemented with further neuropsychological testing, magnetic resonance imaging (MRI) and blood biochemistry testing, as required. Those who passed the MoCA-SG had their case history reviewed by a study neurologist (AN) to minimize the possibility of false-negative MoCA-SG findings. Participants received a diagnosis of no CI, MCI or dementia following the DSM-5 criteria [16]. The overall study protocol is depicted in Figure 1.

### 2.4. Qualitative Interviews

We conducted semi-structured interviews on 10 participants, 1 caregiver and the study psychologist performing VISION-Cog administration (67 ± 12 years, 41.7% female) to supplement quantitative data. The interviews were conducted following the consolidated criteria for reporting qualitative research (COREQ) guidelines, a 32-item checklist designed to improve the transparency and quality of qualitative studies, particularly interviews and focus groups. It covers three key domains: research team reflexivity, study design, and data analysis [19]. The interviews were conducted in-person or by phone following a standardized interview script in their preferred language (English or Mandarin) by a trained interviewer. Responses were recorded, professionally transcribed and analyzed using thematic analysis.

### 2.5. Statistical Analysis

All analyses were conducted using R software version 4.3.1 (R Foundation for Statistical Computing, Vienna, Austria). Participants’ sociodemographic characteristics and medical history, collected using in-house questionnaires; and VISION-Cog completion times, were summarized using mean (SD) for continuous data and proportions for categorical data. Completion rate was defined as the proportion of participants completing the full study protocol of those initially enrolled. Discriminative accuracies of MoCA-SG and VISION-Cog in detecting CI in individuals with VI were assessed using binary logistic regression (exposure: VISION-Cog and MoCA-SG scores analyzed continuously; outcome: presence of CI) with C-statistics to estimate AUC, with the optimal combination of sensitivity and specificity identified using Youden’s Index [20]. Positive and negative predictive values (PPVs and NPVs, respectively) were calculated as (a) the ratio of true positives to all positive results (PPV) and (b) the ratio of true negatives to all negative results (NPV). Confidence intervals were estimated via bootstrapping with 2000 bootstrap replicates in order to minimize the impact of the study’s small sample size. An AUC comparison between the VISION-Cog and MoCA-SG was conducted using Delong’s test, utilizing 2000 bootstrap replications to mitigate issues related to the small sample size, with a two-sided *p*-value < 0.05 considered statistically significant. Sample size calculations were conducted utilizing preliminary AUC data to determine the adequately powered sample size required to identify differences in AUC between the VISION-Cog and MoCA-SG with 80% power and a 5% significance level.

## 3. Results

Of 45 participants recruited, 30 (66.7%) screened positive for CI on MoCA-SG and 15 (33.3%) screened negative. Among those who screened positive, 8 did not attend neurologist evaluation, resulting in 37 participants (82.2%) who completed the full study protocol (22 MoCA-SG-positive and 15 MoCA-SG-negative). Of these 37 participants [mean ± SD age 73.8 ± 6.1 years; mean ± SD near LogMAR VA 0.47 ± 0.14; 19 (51.4%) female; 25 (67.6%) of Chinese ethnicity; Table 1], 25 (67.6%) had ≤10 years of education, 24 (64.9%) were retired and 11 (29.7%) had a monthly income < SGD$1000. Participants who did not attend neurologist evaluation were excluded from statistical analysis.

The overall mean time ± SD for VISION-Cog completion was 59 min 57 s ± 7 min 18 s. Of the 22 participants who failed MoCA-SG and completed the clinical neurologist evaluation, 9 (40.9%) were diagnosed with MCI, with the remainder categorized as cognitively normal. None of the 15 participants who passed MoCA-SG were referred for further neurological evaluation (i.e., were categorized as having normal cognitive ability) after the study neurologist reviewed their case histories (Table 2).

**Figure 2:**
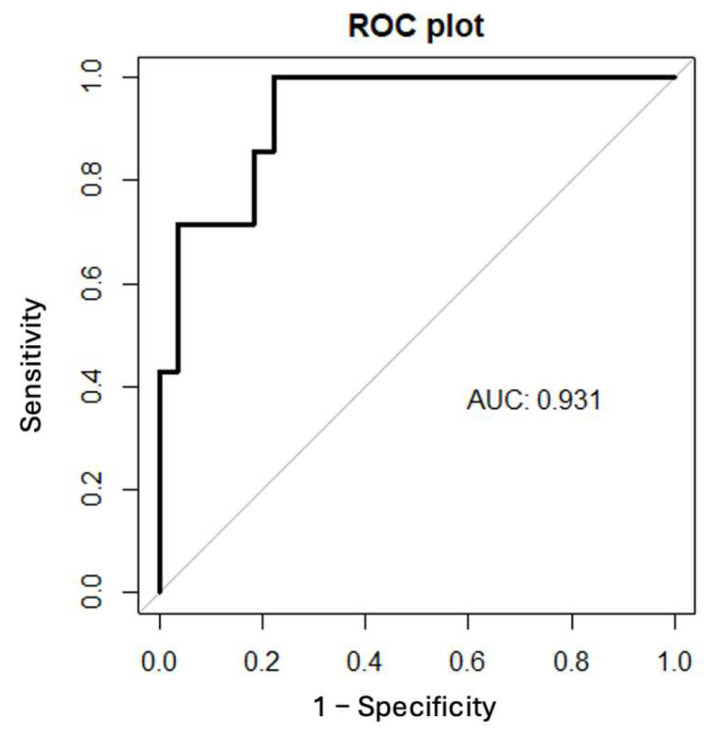
Area under the receiver operating curve (AUROC) for discriminative accuracy of the VISION-Cog as compared to neurologist diagnosis. Legend: ROC—receiver operating curve; AUC—area under the receiver operating curve.

**Figure 3:**
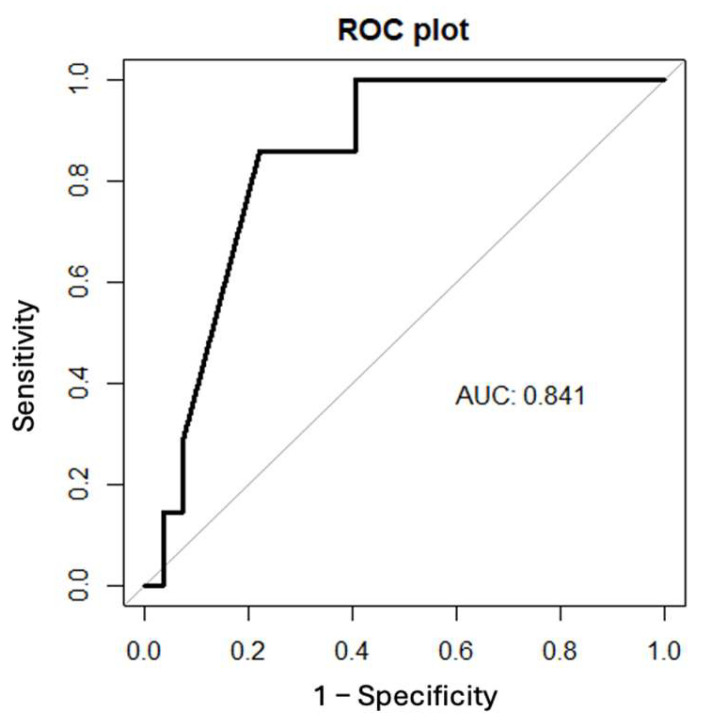
Area under the receiver operating curve (AUROC) for discriminative accuracy of the MoCA-SG as compared to neurologist diagnosis. Legend: ROC—receiver operating curve; AUC—area under the receiver operating curve.

The AUC for VISION-Cog in discriminating between individuals with and without CI in the 37 individuals was 0.93 (95% confidence interval: 0.83, 1.00), with a corresponding sensitivity and specificity of 100.0% (95% confidence interval: 100%, 100%) and 77.8% (95% confidence interval: 59.3%, 92.6%), respectively (Figure 2). The cut-off threshold for discriminating between individuals with and without CI associated with these indices was 233.5 out of maximum 381 points. The PPV and NPV were 0.54 (95% confidence interval: 0.39, 0.79) and 1.00 (95% confidence interval: 1.00, 1.00), respectively. Sensitivity analyses conducted on the 22 participants who failed MoCA-SG revealed slightly lower AUC, sensitivity and specificity indices of 0.85 (95% confidence interval: 0.63, 1.00), 71.4% (95% confidence interval: 42.9%, 100%) and 91.7% (95% confidence interval: 75.0%, 100.0%), respectively. The cut-off threshold for discriminating between individuals with and without CI associated with these indices was 224.5 out of 381 points. The PPV and NPV for these sensitivity data were 0.83 (95% confidence interval: 0.57, 1.00) and 0.85 (95% confidence interval: 0.71, 1.00), respectively. Conversely, while the discriminative accuracy statistics for the MoCA-SG were high overall—0.84 (95% confidence interval: 0.69, 0.96), 85.7% (95% confidence interval: 57.1%, 100.0%) and 77.8% (95% confidence interval: 59.3%, 92.6%) for AUC, sensitivity and specificity, respectively (Figure 3)—these indices were greatly reduced in sensitivity analyses of the 22 participants (AUC: 0.64 [95% confidence interval: 0.38, 0.89], sensitivity: 85.7% [95% confidence interval: 57.1%, 100.0%], specificity: 50.0% [95% confidence interval: 25.0%, 75.0%]). The PPV and NPV for the overall analyses were 0.50 (95% confidence interval: 0.33, 0.79) and 0.96 (95% confidence interval: 0.87, 1.00), respectively, while those for the sensitivity analyses were 0.50 (95% confidence interval: 0.36, 0.70) and 0.86 (95% confidence interval: 0.60, 1.00), respectively. There was no significant difference between the AUCs for the VISION-Cog and MoCA-SG groups (*p* = 0.13).

### 3.1. Sample Size Calculations

Sample size calculations were conducted using the overall AUC of the VISION-Cog (0.93) and MoCA-SG (0.84). To detect a difference of 0.09 AUC with 80% at the 5% significance level, a total sample size of 41 (9 individuals with MCI, 32 cognitively normal persons) was needed.

### 3.2. Qualitative Feedback

Thematic analysis identified two overarching themes from the qualitative feedback: (i) acceptability of modality, with participants expressing that auditory and tactile test formats were intuitive and manageable; (ii) test burden and fatigue, with most participants and the study psychologist noting that, while completion times were acceptable, structured rest intervals at approximately the 30 min mark would improve the testing experience.

## 4. Discussion

This pilot study suggests that the VISION-Cog, a vision-independent neuropsychological test battery, possesses satisfactory preliminary discriminative accuracy for identifying CI in individuals with VI and demonstrates acceptable study completion rates and timing. Larger studies are needed to validate its discriminative accuracy and determine its optimal integration into clinical workflows for CI diagnosis in individuals with VI.

The VISION-Cog’s AUC of 0.931 compares favorably with published values for the standard MoCA in general older adult populations. A 2019 systematic review on MoCA’s ability to discriminate between individuals with and without mild CI and Alzheimer’s disease reported an AUC ranging from 0.71 to 0.99 for mild CI and 0.87–0.99 for Alzheimer’s disease [21]. Importantly, however, these MoCA figures were derived from populations without significant VI. Sensitivity analyses on those who failed the MoCA-SG in our study sample demonstrated marked degradation of the MoCA’s discriminative indices (AUC: 0.643) in those with VI, corroborating the unsuitability of vision-dependent tools in this population. Moreover, the VISION-Cog demonstrated greater specificity indices in sensitivity analyses (91.6%) to detect CI in visually impaired individuals relative to the MoCA-SG (50.0%), suggesting that the vision-neutral design of the former may minimize false-positive CI classification in those with VI. This hypothesis is corroborated by the higher PPV of the VISION-Cog compared to the MoCA-SG in these sensitivity analyses. It is important to note that a higher PPV signifies a reduced false-positive rate. Consequently, VISION-Cog’s enhanced specificity could lead to a considerable reduction in over-referrals to specialist neurological clinics. Given the limited sample size of our study, however, larger studies are necessary to validate the discriminative accuracy of VISION-Cog and elicit specific threshold values for detection of any CI, MCI and dementia.

We acknowledge that the VISION-Cog completion time of 60 min is substantially longer than brief cognitive screeners like the MoCA-SG. However, VISION-Cog was developed as a comprehensive neuropsychological test battery designed to evaluate the five neurocognitive domains critical to CI classification under the DSM-5 criteria. As such, its administration time is more appropriately benchmarked against comparable neuropsychological batteries such as the RBANS [22]. Even though the RBANS has an advertised completion time of 30 min, real-world assessments in older populations with sensory impairments routinely exceed this given timing. The VISION-Cog’s mean completion time of ~60 min is therefore within the range expected of a multi-domain battery in a visually impaired older cohort. This conclusion is supported by qualitative data from our participants that support the acceptability of the VISION-Cog assessment time, although there was consensus to offer participants a break to relieve fatigue. Larger prospective validation cohorts are needed to evaluate whether short rest breaks mid-session can reduce fatigue without substantially affecting the overall procedural duration, and to ascertain the most effective implementation of VISION-Cog within clinical workflows, either as an ancillary procedure in ophthalmic clinics or as a primary diagnostic tool for cognitive impairment in neurological care.

Study strengths include the rigorous clinical examination and comparison with standard neurological diagnoses. Limitations include the small sample size that was not adequately powered to detect statistically significant AUC differences between the VISION-Cog and MoCA-SG. In consideration of the limited sample size, a power analysis performed on our sample of N = 37 participants indicated that we had already attained a statistical power of 76% at the 5% significance level to detect an AUC difference of 0.09 between VISION-Cog and MoCA-SG. Consequently, we do not perceive the sample size limitation to be as severe as initially anticipated. We also did not undertake any comparison of the VISION-Cog with other neuropsychological batteries such as the RBANS. While inclusion of the RBANS was initially considered, it was later abandoned due to concerns over participant burden from completing multiple lengthy tests (VISION-Cog, MoCA-SG, and RBANS). Additionally, the cross-sectional design of this pilot study precludes assessment of VISION-Cog’s longitudinal test–retest reliability or its predictive validity for conversion from MCI to dementia over time. This is an important consideration given that over 46% of individuals with MCI have been reported to progress to clinical dementia within three years. In addition, participants who passed the MoCA-SG did not undergo a full clinical neurological evaluation, which may have led to inflated AUC and associated sensitivity and specificity values. This decision was made out of necessity due to the considerable protocol burden already experienced by participants, as well as the significant backlog faced by clinical neurological teams in Singapore. Lastly, as this was a pilot observational study, formal procedural blinding was not employed. As a result, neurologists were aware of participants’ MoCA-SG scores as the test formed part of the standardized referral protocol and clinical case documentation consistent with real-world standard of care practice in Singapore. While this represents a potential source of diagnostic review bias that we cannot fully exclude, it reflects the pragmatic, real-world design of this pilot study. As such, these pilot data should be interpreted with caution, and formal blinding procedures should be incorporated into future validation studies to mitigate this bias.

In conclusion, the VISION-Cog is an acceptable vision-independent cognitive assessment with good overall diagnostic accuracy (pilot) in identifying CI in VI patients. A larger study is planned to validate its effectiveness against the RBANS and to identify optimal implementation sites and workflows for integration into clinical neurological care.

## Figures and Tables

**Table 1:** Characteristics of study participants (n = 37).

Characteristics	Presented as Mean (SD) or n (%)
Age (years)	73.8 (6.1)
Sex: Female, n (%)	19 (51.4)
Ethnicity, n (%)	
Chinese	25 (67.6)
Malay	2 (5.4)
Indian	10 (27.0)
Education, n (%)	
No formal education	1 (2.7)
Primary	6 (16.2)
Secondary	18 (48.6)
A-level	2 (5.4)
Polytechnic/diploma	3 (8.1)
Vocational training	1 (2.7)
Graduate	2 (5.4)
Post-graduate	4 (10.8)
Monthly household income, n (%)	
<S$1000	11 (29.7)
S$1000 to <S$2000	2 (5.4)
S$2000 to <S$5000	2 (5.4)
S$5000 to <S$10,000	0 (0)
$10,000 or more	5 (13.5)
Do not know	17 (45.9)
Occupation, n (%)	
Professional, Executive & Managerial	3 (8.1)
Housewife	1 (2.7)
Clerical, Administrative	2 (5.4)
Retired	24 (64.9)
Other	7 (18.9)
Near visual acuity, LogMAR	0.47 (0.14)
Cognitive status post-neurologist assessment, n (%)	
Cognitively normal	28 (75.7)
Mild cognitive impairment	9 (24.3)

SD: standard deviation; LogMAR: logarithm of the minimum angle of resolution.

**Table 2:** A 2 × 2 table for the categorization of CI via MoCA-SG and clinical diagnosis (N = 37).

	CI-Positive	CI-Negative
MoCA-SG categorization *	22	15
Neurological diagnosis	9	28 ^†^

* Score < 26 and <27 for individuals with ≤10 years and >10 years of education, respectively. ^†^ Includes 15 individuals who underwent only a medical case history review by a neurologist. CI: cognitive impairment; MoCA-SG: Montreal cognitive assessment test—Singapore version.

## Data Availability

The data presented in this study are available on request from the corresponding author due to Singapore’s privacy restrictions.
